# Biomedical engineering approaches for the delivery of JAGGED1 as a potential tissue regenerative therapy

**DOI:** 10.3389/fbioe.2023.1217211

**Published:** 2023-09-11

**Authors:** Sundus Kaimari, Archana Kamalakar, Steven L. Goudy

**Affiliations:** ^1^ Department of Pediatric Otolaryngology, Emory University, Atlanta, GA, United States; ^2^ Wallace H. Coulter Department of Biomedical Engineering, Georgia Institute of Technology, Atlanta, GA, United States; ^3^ Department of Pediatric Otolaryngology, Children’s Healthcare of Atlanta, Atlanta, GA, United States

**Keywords:** JAGGED1, NOTCH signaling pathway, tissue regenerative therapy, JAGGED1 delivery, biomaterials

## Abstract

JAG1 is a ligand that activates the NOTCH signaling pathway which plays a crucial role in determining cell fate behavior through cell-to-cell signaling. JAG1-NOTCH signaling is required for mesenchymal stem cell (MSC) differentiation into cardiomyocytes and cranial neural crest (CNC) cells differentiation into osteoblasts, making it a regenerative candidate for clinical therapy to treat craniofacial bone loss and myocardial infarction. However, delivery of soluble JAG1 has been found to inhibit NOTCH signaling due to the requirement of JAG1 presentation in a bound form. For JAG1-NOTCH signaling to occur, JAG1 must be immobilized within a scaffold and the correct orientation between the NOTCH receptor and JAG1 must be achieved. The lack of clinically translatable JAG1 delivery methods has driven the exploration of alternative immobilization approaches. This review discusses the role of JAG1 in disease, the clinical role of JAG1 as a treatment, and summarizes current approaches for JAG1 delivery. An in-depth review was conducted on literature that used both *in vivo* and *in vitro* delivery models and observed the canonical versus non-canonical NOTCH pathway activated by JAG1. Studies were then compared and evaluated based on delivery success, functional outcomes, and translatability. Delivering JAG1 to harness its ability to control cell fate has the potential to serve as a therapeutic for many diseases.

## 1 Introduction

JAGGED1 (JAG1) is a ligand that binds to the NOTCH receptor and activates the NOTCH signaling pathway (canonical and non-canonical), as explained in the following section. The NOTCH canonical pathway is involved in determining cellular fate and targeting this pathway can potentially be used as a therapy for diseases that involve JAG1 mutations. Alagille syndrome, for example, is an autosomal dominant disorder that results from nutations in JAG1 and NOTCH2 (Grochowski et al., 2016). Mutations in JAG1 also contribute to heart diseases such as tetralogy of Fallot (TOF), pulmonary stenosis (Bauer et al., 2010), and patent ductus arteriosus (Feng et al., 2010). Additionally, it has been reported that JAG1 overexpression is present in many types of cancer and correlates with a poor clinical prognosis (Xiu et al., 2020). Previous studies have shown that the NOTCH signaling pathway regulates embryological bone formation and mesenchymal cell behavior, such as proliferation and differentiation, *in vitro* and *in vivo* (Engin et al., 2008; Hilton et al., 2008; Zanotti et al., 2008; Mead and Yutzey, 2009; Dong et al., 2010; Salie et al., 2010; Tao et al., 2010; Kohn et al., 2012; Dishowitz et al., 2014). Our lab demonstrated that the NOTCH non-canonical pathway, primarily through JAK-STAT signaling, is required in murine cranial neural crest cell (CNC) osteoblast commitment and mineralization in the presence of canonical NOTCH pathway inhibition (Kamalakar et al., 2021). Previous studies and efforts demonstrated that for proper cell-to-cell signaling, JAG1-NOTCH signaling, and therefore cell differentiation, proliferation, and bone formation to occur, two requirements must be met: immobilizing JAG1 to a delivery scaffold (Vas et al., 2004; Beckstead et al., 2006; Goncalves et al., 2009) and achieving the correct orientation between the NOTCH receptor at the cell surface and the JAG1 ligand of the signaling cell. Obtaining this orientation has been proven to be difficult because JAG1 is a transmembrane protein and therefore signaling is restricted to neighboring cells (Bray, 2006). The use of delivery scaffolds is important because soluble JAG1 has been found to inhibit NOTCH signaling by blocking the available receptors and not allowing Notch intracellular domain (NICD) translocation (Klose et al., 2015). Examples of JAG1 delivery scaffolds that have been used include protein G, polymers such as PolyHEMA, PEG-MAL hydrogels, and poly(β-amino ester), protein G dynabeads, polycaprolatone-incorporated hydroxyapatite (PCL/HA) membranes, self-assembled monolayers, self-assembling peptides, gold nanoparticles, exosomes, PLGA microcarrier beads, and microspheres. All of these approaches will be discussed in detail in Section 5. This review summarizes the delivery mechanisms used for JAG1 as a potential therapy and evaluates the efficacy of these JAG1 delivery methods.

## 2 JAG1 as a NOTCH ligand

**FIGURE 1:**
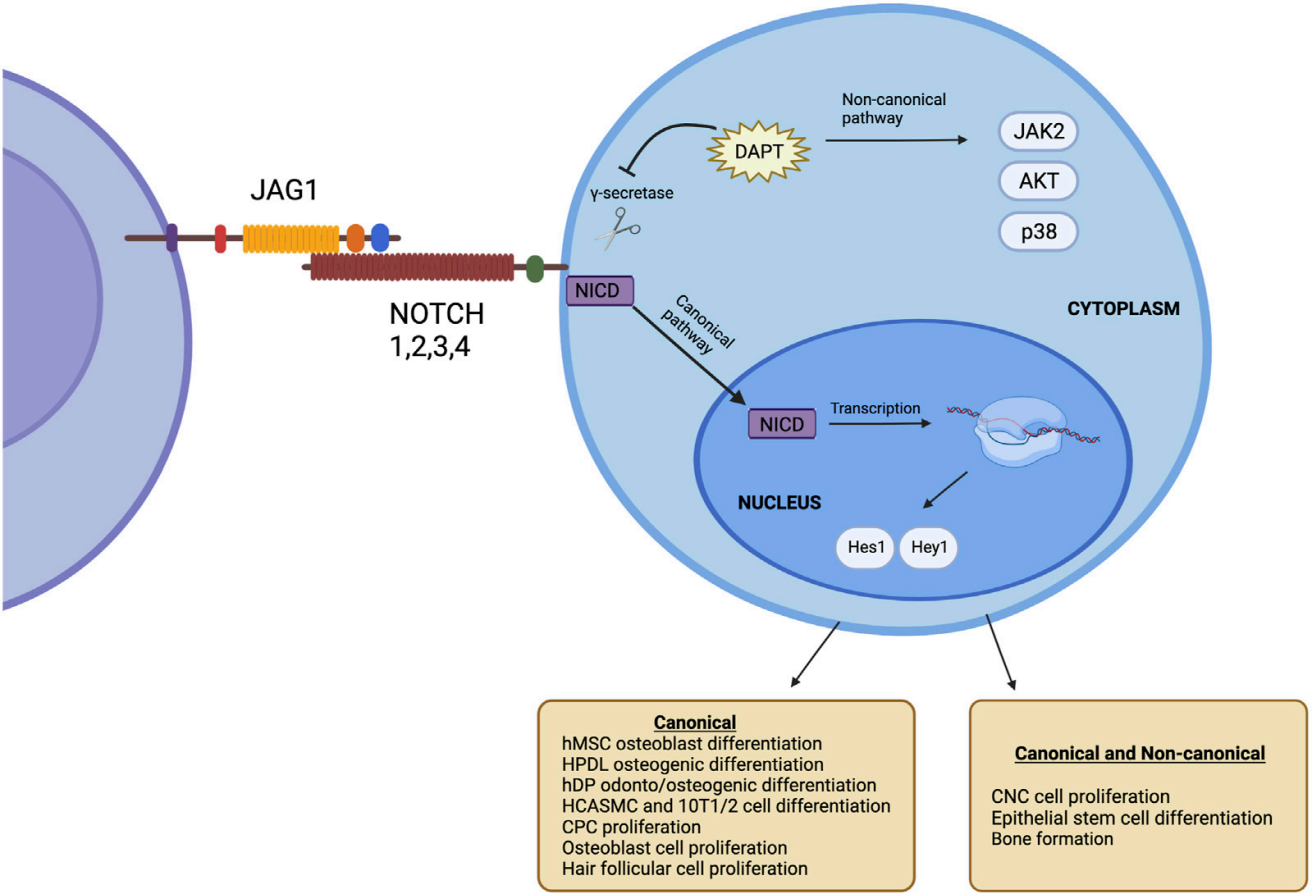
The NOTCH signaling pathway occurs upon the interaction of JAG1 with the NOTCH receptor. This causes **γ**-secretase to cleave the NIC, which moves to the nucleus, leading to the initiation of transcription of NOTCH target genes such as Hes1 and Hey1. This is known as the canonical NOTCH pathway. Target genes are present even after inhibiting **γ**-secretase. This is known as the non-canonical NOTCH. Created with BioRender.com.

JAGGED1 (JAG1) is one of 5 ligands (DLL-1, DLL-3, DLL-4, JAG1, JAG2) that interacts with the NOTCH receptor and therefore plays a key role in NOTCH signaling (Artavanis-Tsakonas et al., 1995). Mammals have 4 NOTCH receptors, known as NOTCH1, NOTCH2, NOTCH3, and NOTCH4, that bind their cognate ligands on the extracellular domain and promote transcriptional regulation at their intracellular domain through canonical and non-canonical signaling (Bray, 2016). NOTCH ligands are bound to the cell surface and bind to NOTCH receptors on adjacent cells through cell-to-cell signaling. JAG1-NOTCH binding leads to the activation of canonical intracellular signaling pathways (Dishowitz et al., 2014). Upon this interaction, the negative regulatory region (NRR) of the NOTCH receptor unfolds and exposes its S2 proteolytic site (Luca et al., 2017), which cleaves the NOTCH intracellular domain (NICD) into the cytoplasm and translocates into the nucleus, resulting in the initiation of transcription of NOTCH target genes such as Hes1 and Hey1 (Dishowitz et al., 2014). This signaling pathway is known as the NOTCH canonical pathway, as illustrated in Figure 1.

**FIGURE 2:**
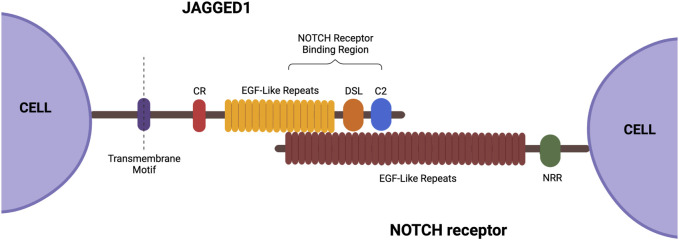
Structure of JAG1. This ligand contains a small intracellular domain and a large extracellular domain. The extracellular domain consists of the following three structural motifs necessary for function: DSL, EGF-like repeats, and a cysteine-rich motif. Created with BioRender.com.

JAG1 is located on chromosome 20, stretching across 36 kb, and has 26 exons ranging in size from 28 to 2,284 bp (Figure 2) (Oda et al., 1997). JAG1 is composed of a small intracellular domain and a large extracellular domain. Generally, the latter domain contains three structural motifs necessary for activation of the NOTCH receptor. At the N-terminal of the protein is a phospholipid-binding C2 domain that contributes to NOTCH activation by providing both cell membrane and NOTCH interaction sites. A study done by Meng et al. (2022) found that N-glycosylation of this C2 domain is required for efficient phospholipid binding and NOTCH activation by JAG1. This is because the N-glycan promotes the required orientation of JAG1 for NOTCH signaling. C terminal to this domain is the 40 amino acid DSL (Delta/Serrate/LAG-2) motif which is required for the binding of JAG1 to the epidermal growth factor-like (EGF) repeats of the NOTCH receptors. The second motif consists of the 16 EGF repeats and they play a vital role in increasing JAG1’s affinity to the NOTCH receptors (Bray, 2006; Luca et al., 2017). This is followed by a cysteine-rich (CR) motif, which is only present in JAG1 and JAG2 (Luca et al., 2017). The CR motif contributes to protein stability and protein-protein interactions (Grochowski et al., 2016).

## 3 The role of JAG1 in disease

### 3.1 Alagille syndrome (ALGS)

Heterozygous mutations in JAG1 (Colliton et al., 2001) and/or mutations in NOTCH1 (Li et al., 1997) and NOTCH2 (Kamath et al., 2012) result in the autosomal-dominant disorder Alagille syndrome (ALGS). This disorder is characterized by abnormalities in the heart, skeleton, eye, face, and bile ducts, which leads to liver failure (Colliton et al., 2001). The phenotype of this disorder is highly variable from one patient to another and clinical diagnosis has been based on observing the paucity of the intrahepatic bile ducts on a liver biopsy in association with at least 3 of 5 major clinical features: chronic cholestasis, cardiac disease (primarily peripheral pulmonary artery stenosis), facial features (mild dysmorphic features including maxillary hypoplasia), skeletal abnormalities (butterfly vertebrae), and ocular abnormalities (primarily posterior embryotoxon) (Colliton et al., 2001; Kamath et al., 2003; Turnpenny and Ellard, 2012). JAG1 mutations in ALGS range from total gene deletions to prominent protein truncating mutations (85% of cases) such as nonsense, missense, splicing, and gene deletion mutations (Colliton et al., 2001). Mutation analysis of JAG1 in Alagille syndrome patients has shown that mutations occur adjacent to the DSL region of JAG1 (Colliton et al., 2001). Studies have sought to understand the role of JAG1 in development and disease. A study done by Hayashi et al. generated and characterized two homozygous JAG1 gene knockout iPSC lines and observed that JAG1 protein expression was compromised in these lines, making them a promising means to study the role of JAG1 in human development and pathology (Song et al., 2021). However, there is very little evidence for genotype-phenotype correlation in ALGS and JAG1, suggesting that identifying a JAG1 mutation cannot predict the severity of the disease (Kamath et al., 2003).

### 3.2 Cardiovascular diseases

Tetralogy of Fallot (TOF) is a prevalent congenital heart defect with a rate of 3.3 cases per 10,000 live births and it affects normal blood flow through the heart (Bauer et al., 2010). Pulmonary stenosis is the narrowing of the pulmonary valve which controls blood flow from the heart’s right ventricle into the pulmonary artery. It has been established that NOTCH signaling plays an important role in cardiac development (de la Pompa, 2009) and previous studies have reported mutations in JAG1 that caused cardiac defects in non-syndromic individuals (Krantz et al., 1999; Eldadah et al., 2001; Le Caignec et al., 2002; Bauer et al., 2010). A 2010 study screened a cohort with right-sided cardiac defects for JAG1 mutations and found functionally significant sequence variants in 3% of the TOF cases and 4% of the pulmonic stenosis/peripheral pulmonary stenosis/pulmonary valve atresia with intact ventricular septum cases. These cases were confirmed to not meet the criteria for ALGS diagnosis and did not have any other syndromes (Bauer et al., 2010). To understand the role of JAG1 in cardiovascular disease, efforts have been made to delete JAG1 during cardiac development and observe the impact. In one study, JAG1 was removed from the craniofacial mesenchyme to demonstrate its role in palatal arterial development. Upon harvesting the mutant mice embryos, it was found that they had a hypoplastic osseous palate and irregularly spaced palatal rugae (Humphreys and Goudy, 2008). The primary role of endothelial JAG1 in potentiating the development of neighboring vascular smooth muscle was highlighted in a study by High et al. (2008). They demonstrated that endothelial specific deletion of JAG1 resulted in embryonic lethality associated with cardiovascular defects. Moreover, expression of smooth muscle markers was diminished in the endothelial-specific JAG1 mutant embryos. In another study, High et al. (2009) deleted JAG1 or inhibited NOTCH signaling in the second heart field. This led to the downregulation of the following critical factors involved in outflow tract development: Fgf8 and bone morphogenetic protein 4. Deletion of JAG1 in murine vascular smooth muscle cells was attempted by Feng et al. (2010) and it was reported that early postnatal mortality was observed in these mice due to patent ductus arteriosus, which is a common congenital heart defect. This defect occurs when the ductus arteriosus, an arterial vessel that shunts blood flow away from the lungs during fetal life, is not closed after birth. Manderfield et al. (2012) deleted JAG1 in neural crest cells and results demonstrated that vascular smooth muscle differentiation was impaired and led to aortic arch artery defects. JAG1 mutant mice embryos were studied by Robert-Moreno et al. (2008) to understand the role of JAG1 in embryonic hematopoiesis. It was found that hematopoiesis was altered in JAG1 mutants compared to the JAG2 mutants and the control. Moreover, the expression of the NOTCH marker GATA2 was compromised in the JAG1 mutants. These results suggest that JAG1 is responsible for activating NOTCH in hematopoietic cells in the dorsal aorta and that JAG is required for embryonic hematopoietic development *in vivo*.

### 3.3 Cancer

The role of the NOTCH signaling pathway and JAG1 in different types of cancer development is well established given their involvement in cell proliferation, differentiation, apoptosis, and angiogenesis (Miele et al., 2006; Luo et al., 2017). It has been reported that JAG1 overexpression is present in many types of cancer and correlates with a poor clinical prognosis (Xiu et al., 2020). Previous studies have established this by demonstrating that JAG1 is highly expressed in metastatic prostate cancer (Santagata et al., 2004; Pedrosa et al., 2016) and that high-level JAG1-NOTCH expression is observed in the angiogenesis of breast cancer (Reedijk et al., 2005). Additionally, the presence of JAG1 and NOTCH3 in triple negative breast cancer has been established (Xue et al., 2017). A recent study investigated the mechanism that mediates the crosstalk between NOTCH and STAT3 pathways in platinum-resistant ovarian cancer and found that STAT3 and JAG1 are overexpressed in such cancer tissues (Yang et al., 2019). Pedrosa et al. (2015) was the first study to investigate the role of endothelial JAG1-mediated NOTCH signaling in tumor angiogenesis. Tumor angiogenesis occurs when endothelial cells migrate towards the growing mass when responding to local stimuli. In this study, two mouse tumor models were used: subcutaneous Lewis Lung Carcinoma (LLC) tumor transplants and the autochthonous Transgenic Adenocarcinoma of the Mouse Prostate (TRAMP). Endothelial-specific JAG1 gain and loss-of-function mutants we used to understand the role of JAG1 in tumor growth and angiogenesis. It was concluded that modulating levels of endothelial JAG1 had an inhibitory effect in angiogenic and maturation responses for both tumor models. JAG1 was also found to be upregulated by SOX12 in osteosarcoma tumor growth (Zhang et al., 2021). In 2017, a study measured protein expression of JAG1 and DLL4 in adenocarcinomas (AC) and squamous cell/adeno-squamous carcinomas (SC/ASCs) to establish a targeted therapy for gallbladder cancers. The study found that positive JAG1 and DLL4 expression is associated with clinicopathological characteristics of gallbladder cancers (Luo et al., 2017). JAG1 has also been implicated in leukemia (De Falco et al., 2018). Given findings such as these have made JAG1 inhibitory therapies an attractive target for cancer therapy in these tumors.

## 4 Clinical applications of JAG1

Despite its role in disease and cancer, JAG1 has the potential to be a therapeutic molecule for many diseases due to JAG1’s role in cellular proliferation and differentiation. For example, the potential role of JAG1 as a bone regenerative therapy to combat bone loss and frequent bone fractures in ALGS (Youngstrom et al., 2016), and craniofacial bone loss or low bone mineral density (BMD) has been implicated (Davis-Knowlton et al., 2019). In a study that investigated the expression of NOTCH signaling during osteogenic differentiation *in vitro* and *in vivo*, JAG1 was upregulated in both *in vitro* osteogenic differentiation and *in vivo* tibial bone fracture healing. Moreover, JAG1 activated NOTCH signaling in human alveolar and iliac bone-derived cells resulted in enhanced mineralization *in vitro* (Osathanon et al., 2019). NOTCH canonical signaling by JAG1 has also been found to regulate mesenchymal stem cells (MSC) osteoblast commitment and differentiation (Zhu et al., 2013; Dishowitz et al., 2014; Youngstrom et al., 2017; Ndong et al., 2018). In addition to the NOTCH canonical pathway, the role of JAG1 in inducing the differentiation of CNC cells to osteoblasts via a novel non-canonical NOTCH pathway has been established (Kamalakar et al., 2021).

JAG1 may also be applied in dentistry. It has been shown that JAG1 enhances osteogenic differentiation of human periodontal ligament derived mesenchymal stem cells (HPDLs) through NOTCH signaling which can potentially be used as a therapeutic in periodontal defects (Osathanon et al., 2013; Nowwarote et al., 2018). Additionally, immobilization of JAG1 induced odonto/osteogenic differentiation in human pulp cells (hDPs) suggests its potential role in reparative dentin formation (Manokawinchoke et al., 2017; Manaspon et al., 2020). In another study that further confirms the role of JAG1 in hDP differentiation, Kornsuthisopon et al. (2021) described the participation of the cytokine IL-15 in JAG1-induced mineralization in hDPs. The study demonstrated that cells treated with JAG1 resulted in the upregulation of IL-15 and IL-15RA mRNA expression and cells treated with IL-15 resulted in a significant increase in mineral deposition. Hansamuit was another study that defined the effect of JAG1 on hDP differentiation. In this study, hDPs expressed higher NOTCH target genes (HES1 and HEY1) when they were seeded on immobilized JAG1 (Hansamuit et al., 2020). A study done by Kornsuthisopon et al. (2022) also used hDPSCs to demonstrate the crosstalk between the NOTCH and non-canonical Wnt signaling pathways. The study found that treating hDPSCs with JAG1 and the Wnt ligand WNT5A promoted their mineralization.

The clinical roles of JAG1 can also extend to addressing cardiac conditions such as myocardial infarction and improving cardiac development. It has been demonstrated that JAG1-NOTCH signaling promotes the differentiation of MSC into cardiomyocytes which helps improve left ventricular function (Li et al., 2006). Delivery of a peptide mimic of JAG1 to an infarcted rat heart was shown to promote the differentiation of cardiac progenitor cells (CPC) into cardiomyocytes and improve cardiac function and contractility, suggesting the ability of JAG1 to serve as a regenerative response following myocardial injury (Boopathy et al., 2014; Boopathy et al., 2015). Vascular development, including angiogenesis, is another clinically-relevant role of JAG1 during embryogenesis and wound repair. An increase in the formation of newly generated vessels upon delivering JAG1, using a Matrigel plug assay, to the flanks of athymic nude mice has been observed (Gonzalez-King et al., 2017). Atherosclerosis has been directly linked to NOTCH dysregulation therefore making NOTCH signaling a target for developing therapeutics to treat coronary artery disease (Aquila et al., 2017; Davis-Knowlton et al., 2019; Zohorsky et al., 2021). It has been demonstrated that the delivery of JAG1 controls the phenotype of primary human coronary artery smooth muscle cells and promotes the vascular differentiation of progenitor cells (Zohorsky et al., 2021). Moreover, the potential of JAG1 as a target for tumor angiogenesis was demonstrated, where it was found that an increase in endothelial JAG1 expression led to an increase in tumor size (Pedrosa et al., 2015).

Hair loss is another clinical problem JAG1 addresses as a study has shown its therapeutic potential for androgenetic alopecia. The topical application of JAG1 promoted hair regeneration and its effect was enhanced when combined with epidermal growth factor (Lin et al., 2019). In another study that highlights the role of JAG1 in hair growth, Estrach et al. (2006) aimed to explain how the Wnt and NOTCH signaling pathways interact to regulate hair follicle maintenance. They found that the deletion of JAG1 in mice resulted in the inhibition of the hair growth cycle and the conversion of hair follicles into cysts of cells undergoing interfollicular epidermal differentiation. Moreover, blocking NOTCH signaling through JAG1 deletion prevented the induction of new hair follicle formation by the Wnt target, ß-catenin, in the epidermis of adult mice. JAG1-NOTCH signaling was highlighted in a study that aimed to demonstrate the role of T regulatory cells (Treg) in hair follicle stem cell (HFSC) biology. Through transcriptional and phenotypic profiling of Tregs and HFSCs, it was revealed that skin-resident Tregs express high levels of JAG1. The interaction of the expressed JAG1 from the Treg cells with the NOTCH receptors of the HFSCs facilitated HFSC function and efficient HF regeneration. Therefore, it was concluded that Treg expression of JAG1 is required for efficient hair regeneration (Ali et al., 2017).

## 5 Delivery methods

**FIGURE 3:**
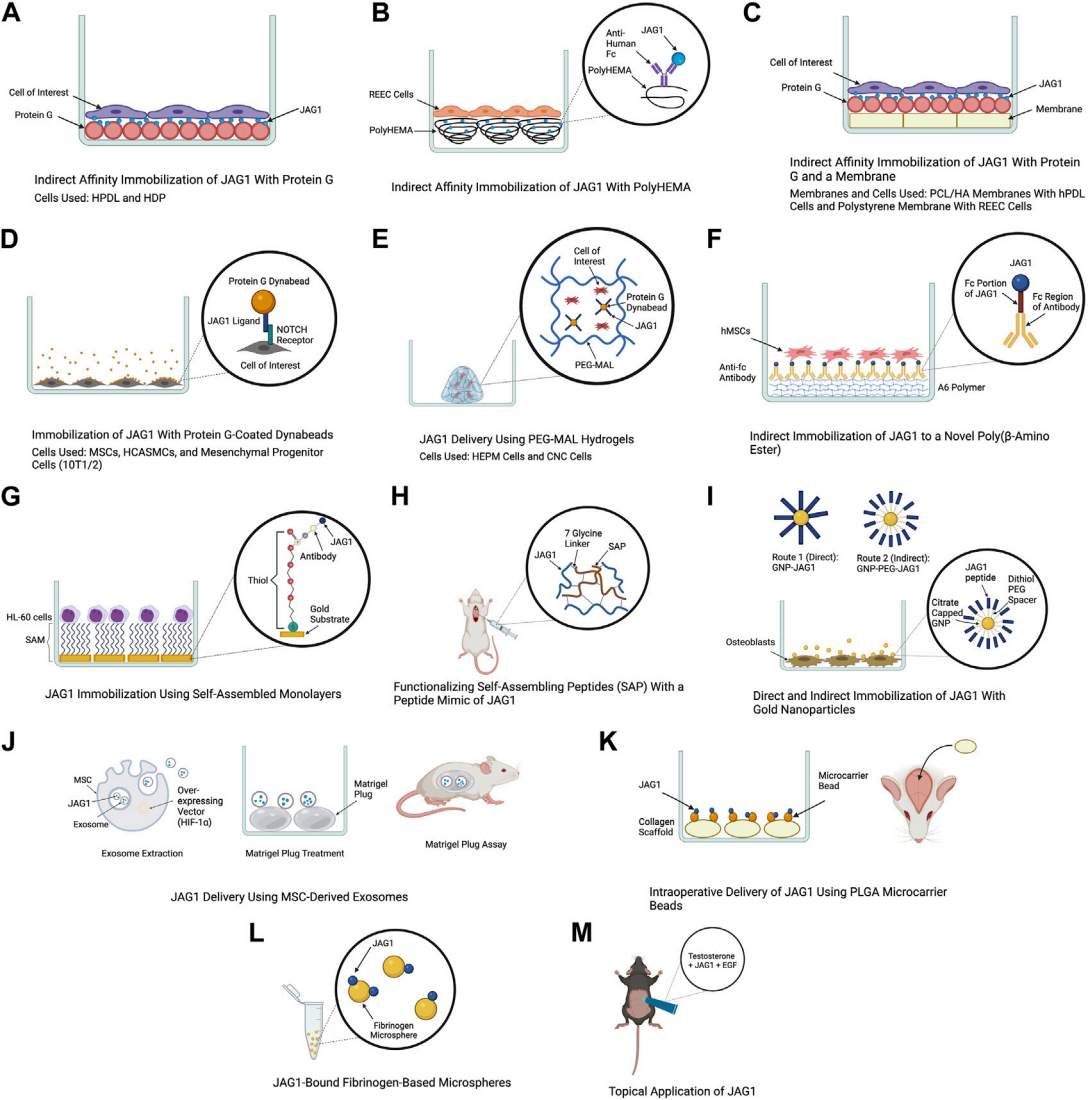
Delivery methods of JAG1. The clinical applications of the JAG1-NOTCH signaling pathway as well as the difficulty of delivering JAG1 has led to the development of different immobilization strategies. Created with BioRender.com.

The role of JAG1-NOTCH signaling in regulating cell behavior such as differentiation, proliferation, and apoptosis has been widely researched. Delivery of JAG1 requires the protein to be bound or immobilized in a way that the receiving cell’s NOTCH receptor will be cleaved into NICD. Delivering soluble JAG1 actively inhibits NOTCH signaling by occupying the receptor but does not lead to NICD cleavage. Therefore, the immobilization of JAG1 to a delivery scaffold is required and is a significant hurdle to developing JAG1-based therapies. Delivering JAG1 in the correct orientation to signal with the NOTCH receptor at the cell surface is critical for NOTCH signaling (Wang, 2011). Taking these requirements into consideration, studies have attempted to deliver JAG1 as a therapeutic and are summarized in Table 1 and Figure 3.

**TABLE 1:** Delivery strategies for JAG1.

Delivery/Immobilization method	Test subject	Study type	NOTCH pathway observed	Studies
Indirect affinity immobilization with protein G	HPDL cells	*In vitro*	Canonical	Osathanon et al. (2013)
	hDP cells	*In vitro*	Canonical	Manokawinchoke et al. (2017)
	hDPSCs	*In vitro*	Canonical	Chansaenroj et al. (2022)
Indirect affinity immobilization with protein G and membrane	REEC cells with polystyrene membrane	*In vitro*	Canonical and non-canonical	Beckstead et al. (2006)
	HPDL cells with PCL/HA membranes	*In vitro*	Canonical	Nowwarote et al. (2018)
Indirect affinity immobilization with polyHEMA	REEC cells	*In vitro*	Canonical and non-canonical	Beckstead et al. (2006)
Immobilization of JAG1 with Protein G-coated dynabeads	Primary human and mouse MSCs	*In vitro*	Canonical	Zhu et al. (2013)
	HCASMCs and 10T1/2 cells	*In vitro*	Canonical	Zohorsky et al. (2021)
JAG1 delivery using PEG-MAL hydrogels	HEPM cells	*In vitro*	Canonical	Ndong et al. (2018)
	CNC cells and C57BL/6 mice	*In vitro*	Canonical and non-canonical	Kamalakar et al. (2021)
Indirect immobilization of JAG1 to a novel poly(β-amino ester)	hMSCs	*In vitro*	Canonical	Dishowitz et al. (2014)
JAG1 immobilization using self-assembled monolayers	HL-60 leukemic cell line	*In vitro*	Canonical	Goncalves et al. (2009)
Functionalizing self-assembling peptides with a peptide mimic of JAG1	CPCs and Rats	*In vitro* and *in vivo*	Canonical	Boopathy et al. (2014)
	Female adult Sprague Dawley rats	*In vivo*	Canonical	Boopathy et al. (2015)
Direct and indirect immobilization of JAG1 with gold nanoparticles	MC3T3-E1 osteoblast cell line	*In vitro*	Canonical	Matea et al. (2015)
JAG1 encapsulated within MSC-derived exosomes	MSCs and Athymic-nude (nu/nu) mice	*In vivo*	Canonical	Gonzalez-King et al. (2017)
Intraoperative delivery of JAG1 using PLGA microcarrier beads	Wild-type C57BL/6 mice and Sprague Dawley rats	*In vivo*	Canonical	Youngstrom et al. (2017)
Binding JAG1 to fibrinogen-based microspheres	hDP cells	*In vitro*	Canonical	Manaspon et al. (2020)
Topical application of JAG1	Eight-week-old male C57BL/6 mice	*In vivo*	Canonical	Lin et al. (2019)

The use of protein G is a common method for JAG1 immobilization as it is a protein that binds immunoglobulin G (IgG) of many species. Additionally, commercially produced JAG1 contains a tag protein, such as a Fc fragment that protein G can interact with, which helps orient JAG1. Earlier studies have used an *in vitro* indirect affinity immobilization method to immobilize JAG1, where surface-bound JAG1 was prepared by incubating the wells of a tissue culture plate with recombinant protein G followed by incubation with JAG1 and the desired cell line as shown in Figure 3A (Osathanon et al., 2013; Manokawinchoke et al., 2017). Osathanon et al. (2013) used this method to investigate the ability of JAG1 to control the differentiation of HPDLs. Incubating JAG1 with HPDLs resulted in downregulation of negative regulators of osteogenic differentiation, and key osteogenic differentiation factors were examined. Manokawinchoke et al. (2017) also used this immobilization method to investigate the response of hDPs to JAG1 *in vitro*. It was found that JAG1-treated hDPs exhibited downregulation of genes related to the cell cycle and DNA replication and immobilized JAG1 promoted odonto/osteogenic differentiation in hDPs. Beckstead et al. (2006) used this method to examine the role of NOTCH signaling in esophageal epithelial differentiation where two biomaterial surfaces were tested for JAG1 immobilization. The first surface tested was a hydrophilic negatively charged polystyrene. Recombinant protein G was precoated to this surface and this was then incubated with Jagged-1/Fc fusion protein. PolyHEMA was the second biomaterial used for JAG1 indirect affinity immobilization (Figure 3B). This is a commonly used biomaterial due to its resistance to protein adsorption, therefore providing a blank for covalent immobilization studies. The polyHEMA was spin cast on glass coverslips and anti-human Fc was covalently bound to it. JAG-1/Fc was then bound to this complex. Both biomaterials were then incubated with rat esophageal epithelial cells (REEC). Both JAG1-treated biomaterial surfaces were tested for NOTCH activation and it was found that two NOTCH target genes (*caspase 3* and *occludin*) were upregulated compared to the control groups (no treatment and Fc control). Additionally, endothelial cell differentiation by these biomaterials was confirmed by observing an increase in intermediate (involucrin and CK10) and late (filaggrin) stage differentiation markers. Nowwarote et al. (2018) used this same immobilization method to develop membranes that can be used as an alternative guided tissue regeneration membrane to promote osteogenic differentiation in periodontal defects (Figure 3C). PCL/HA membranes coated with JAG1 were incubated with recombinant protein G and the response of hPDLs on these membranes were evaluated *in vitro*. The results demonstrated that indirect affinity immobilized JAG1 on the membranes upregulated hPDL NOTCH target gene expression. Recently, Chansaenroj et al. (2022) also used this immobilization method to understand the effect of JAG1 and the extracellular matrix (ECM) on the osteogenic differentiation of hDPSC. In this study, surfaces were incubated with protein G, human IgG Fc fragment, and rhJagged1/Fc recombinant protein. The hDPSCs that were exposed to osteogenic differentiation culture conditions were subsequently seeded on these surfaces. Upon performing a mineralization assay, it was found that the indirect immobilized JAG1 stimulated a membrane trafficking protein (DOP1B) that indirectly enhances osteogenic differentiation. Additionally, proteins from hDPSCs were extracted and subcellular components were analyzed. It was found that hDPSCs cultured with JAG1 under osteogenic differentiation conditions expressed the following matrisome proteins (COL27A1, MXRA5, COL7A1, and MMP16), which play a role in osteogenic differentiation. These results demonstrate the interplay of JAG1 and ECM on osteogenic differentiation.

The use of super paramagnetic spherical beads called dynabeads, coated with protein G is another approach to immobilize JAG1 (Figure 3D). For example, a study that aimed to understand the effect of JAG1-activated NOTCH signaling on MSC osteoblast differentiation used agarose protein G beads to immobilize JAG1. Primary human and mouse MSCs were treated with JAG1 *in vitro* and upon evaluation, it was found that JAG1 induced differentiation of human MSC into osteoblasts through canonical NOTCH signaling while osteoblastogenesis was inhibited in mouse MSCs demonstrating a major pitfall in the use of mouse models to study bone abnormalities for diseases such as ALGS (Zhu et al., 2013). To develop a strategy to deliver JAG1 to control vascular tone and vascular differentiation in the absence of an endothelial cell signal, Zohorsky et al. (2021) also used protein G-coated dynabeads to immobilize JAG1. Human coronary artery smooth muscle cells (HCASMC) and embryonic multipotent mesenchymal progenitor (10T1/2) cells were treated with Dynabead-bound JAG1 and NOTCH activation, differentiation, and maturation of these cells was observed. Protein G-coated dynabeads were also used to immobilize JAG1 in a study that aimed to evaluate the osteogenic potential of the NOTCH canonical pathway *in vitro*. As shown in Figure 3E, JAG1 delivery was done by encapsulating the JAG1-bound dynabeads and human embryonic palatal mesenchyme (HEPM) cells in polyethylene glycol maleimide (PEG-4MAL) hydrogels. The results of this study demonstrated increased alkaline phosphate (ALP) activity and osteoblast gene expression compared to controls, indicating that the JAG1-PEG-MAL complex can induce HEPM cell commitment to osteoblast cell fate. Moreover, this study established a proof of principle for *in vivo* applications (Ndong et al., 2018). A study that aimed to characterize the osteo-inductive properties of JAG1 and harness its potential as a bone regenerative therapy used this same immobilization method. However, PEG-4MAL hydrogels were used to encapsulate the JAG1-bound dynabeads as well as CNC cells. These hydrogels were then implanted within critically-sized parietal bone defects in mice and the results demonstrated that the JAG1-PEG-4MAL-CNC hydrogels improved closure of the cranial defect (Kamalakar et al., 2021).

Other polymers have been developed to be used for JAG1 immobilization. A study that aimed to develop a clinically translatable therapy for bone regeneration immobilized JAG1 to a novel poly(β-amino ester) comprised of diethylene glycol diacrylate (A) and isobutylamine (6), known as A6 (Figure 3F). This polymer was used due to its osteoconductive capability as a scaffold for protein delivery. To synthesize A6 for the *in vitro* experiments, A6 and the photoinitiator 2,2-dimethoxy-2-phenylacetophenone (DMPA) were mixed with an equal amount of ethanol. This mixture was then used to coat the wells of a 24-well tissue culture plate. Finally, the A6 macromer was photopolymerized by exposure to ultraviolet light in a nitrogen-purged environment. This study explored direct and indirect immobilization to establish an optimal immobilization strategy that induces NOTCH activation at higher protein concentrations which is required for *in vivo* therapeutic effects. For direct immobilization, JAG1 was diluted with PBS at various concentrations and was adsorbed to A6, whereas for indirect immobilization, a secondary antibody (anti-Fc antibody) was adsorbed to A6 and the anti-Fc antibody was in turn bound to the Fc portion of JAG1. Upon immobilizing JAG1 and evaluating it in hMSCs, the results demonstrated that JAG1 induced an osteogenic phenotype in hMSCs, with the direct strategy being more effective (Dishowitz et al., 2014).

Self-assembled monolayers (SAMs) is another approach that has been used for JAG1 immobilization. SAMs are highly ordered organic substrates obtained by spontaneous organization of alkanethiols on surfaces. Some features that make these surfaces favorable over other substrates for immobilization is that they provide precision of surface control, different functional groups can be exposed on the same surface, and they allow a broad range of surface chemistry modifications (Goncalves et al., 2009). One of the earlier studies that established the use of SAM as a strategy for JAG1 immobilization was done in 2009 where the study aimed to design a surface at the nanoscale level that induces NOTCH signaling (Figure 3G). The study used gold substrates to assemble the SAM and immobilized an antibody to it to facilitate JAG1 immobilization. A leukemic cell line (HL-60) was treated with this JAG-1-Ab-SAMs complex and it was observed that this surface could induce different levels of NOTCH signaling activation (Goncalves et al., 2009).

Functionalizing self-assembling peptides (SAP) with a peptide mimic of JAG1 has also been used as a therapeutic to deliver JAG1. Boopathy et al. (2014) used this approach to evaluate the therapeutic benefits of CPC delivery in a myocardial infarction rat model (Figure 3H). The SAP used in this study was generated with a 7-glycine linker followed by JAG1. It had hydrophilic and hydrophobic residues with alternating charges that allowed for self-assembly into nanofiber hydrogels at physiologic pH and osmolarity. Improvement in acute retention and cardiac function was observed in rats subjected to experimental myocardial infarction upon cell therapy using these hydrogels. Following this study, Boopathy et al. (2015) used the same delivery method to examine these SAP hydrogels in a cell-free setting and evaluate their effect on the endogenous healing response following myocardial infarction. It was found that delivery of the peptide mimic of JAG1 in the cell-free SAP hydrogel to the infarcted rat heart resulted in improved cardiac function and decreased fibrosis.

Another method for JAG1 delivery is functionalizing nanocarriers with JAG1. Matea et al. (2015) aimed to develop a route to functionalize gold nanoparticles (GNPs) with JAG1 to promote an efficient interaction between JAG1 and the NOTCH receptor. Two routes were developed and the resulting nanostructures were evaluated in terms of functionality and toxicity. As illustrated in Figure 3I, the first route proposed to couple JAG1 directly on the GNPs surface through the thiol functional groups from the cysteine residues present in JAG1 (GNP-JAG1) while the second route coupled JAG1 to a dithiol PEG spacer that was coupled with the GNPs surface (GNP-PEG-JAG1). Upon exposing osteoblasts to different concentrations of PEGylated nanostructures, no significant change in their viability and proliferation rate was observed. However, higher concentrations of the nanostructures decreased the proliferation rate, while lower concentrations promoted cell proliferation. Moreover, it was demonstrated that the presence of a PEG spacer between JAG1 and the gold core resulted in identical (monodispersed), aqueous stable nanostructures while GNP-JAG1 nanostructures were non-identical (polydispersed). A monodispersed distribution of these nanoparticles is ideal because achieving homogeneity in size and shape ensures consistency in JAG1 delivery.

Exosomes are extracellular vesicles that are released from cells when the endocytic compartment, the multivesicular body, fuses with the plasma membrane. They have been implicated in cell-cell communication as they contain biological material such as mRNAs, miRNAs, proteins, lipids, and most importantly NOTCH ligands that can directly stimulate target cells. Moreover, studies have demonstrated exosome-mediated induction of functional activity in target cells (Hoshino et al., 2015; Edgar, 2016; Marino et al., 2016). These characteristics of exosomes were harnessed in a study that packaged JAG1 in MSC-derived exosomes to induce JAG1-mediated angiogenesis (Figure 3J). Exosomes were derived from human dental pulp MSCs that were transfected with a hypoxia inducible factor-1α (HIF-1α) overexpressing vector (HIF-MSC), which has been shown to induce JAG1 expression and exosome secretion. To assess the effect of the exosome on neovascularization *in vivo*, a Matrigel plug assay was performed where the JAG1 containing exosomes were incubated with a Matrigel plug, which was placed in athymic nude mice. The results demonstrated an increase in formation of new vessels on the gel, that JAG1 was the only NOTCH ligand present in the HIF-MSC exosomes, and HIF-1α increased the levels of JAG1 both at the cellular and exosome level (Gonzalez-King et al., 2017).

The delivery of JAG1 using clinically applicable collagen scaffolds to serve as a therapy for skeletal dysfunction was performed in a study done by Youngstrom et al. (2017) as illustrated in Figure 3K. JAG1 was first encapsulated and delivered to calvarial defects in mice via collagen grafts. These grafts were incubated with recombinant human JAG1 on 25 μg PLGA microcarrier beads in PBS to examine if JAG1 could promote bone regeneration *in vivo*. Gelfoam grafts were then used to bind and deliver JAG1 to a mouse femoral defect to determine whether it could increase bone regeneration in the appendicular skeleton. Finally, collagen hemostatic sponge grafts were functionalized with recombinant human JAG1 and placed within rat calvarial defects to determine if JAG1 could promote regeneration in a species other than mice. Regeneration occurred in all three cases suggesting that direct delivery of recombinant JAG1 to bone injuries could be a therapeutic approach for skeletal regeneration.

Manaspon et al. (2020) emphasized the importance of immobilizing JAG1 in their study where they aimed to suggest a JAG1 delivery system that transports immobilized JAG1 and promotes odontogenic differentiation of human dental pulp cells. This was done by preparing two types of microspheres as biomaterials for JAG1 immobilization and delivery (Figure 3L). Fibrinogen microspheres (FbMs) were prepared using a water and oil emulsification method where these microspheres were spontaneously formed upon adding the fibrinogen solution in oil. The thrombin crosslinked fibrinogen microspheres (tFbMs) were prepared by the same means, however, the addition of thrombin served to enhance and develop interconnection networks between fibrinogens in the matrix. JAG1 was conjugated on these microspheres using EDC/NHS cross-linking where the C-terminus of JAG1 was crosslinked with the N-terminal of fibrinogen. It was found that the binding capacity of JAG1 on the both microspheres were within the same range. Cell viability and cytotoxicity of these microspheres against hDPS were also evaluated and it was concluded that there was no significant change in cell viability after the hDPs were exposed to various concentration of FbM and tFbM extracted medium.

Harnessing JAG1 as a therapeutic is not necessarily limited to being delivered in the body. For example, Lin et al. (2019) demonstrated the therapeutic potential of JAG1 for androgenetic alopecia by applying it topically to adult male mice (Figure 3M). Topical application was performed by dissolving JAG1 with 0.5% of testosterone and applying this solution to dorsal depilatory mice. It was found that JAG1 had a hair growth stimulating effect, however, this effect was enhanced when it was used in combination with EGF, suggesting a synergistic effect of EGF and JAG1 in an androgen-suppressed hair regrowth model *in vivo*.

## 6 Discussion

Activation of the NOTCH pathway by JAG1 has a broad range of clinical roles and its therapeutic potential is evident in many studies. However, there is a challenge to find an optimal method that safely delivers this ligand and guarantees the activation of the NOTCH pathway. For it to be clinically applicable and prevent its soluble form from inhibiting NOTCH signaling, it must be immobilized and it must be in a correct orientation with the NOTCH receptor to be able to activate the NOTCH signaling pathway upon delivery. Despite a number of papers demonstrating the efficacy of binding JAG1 with various biomaterials and scaffolds, some issues remain unaddressed. Indirect affinity immobilization of JAG1 is a common method for JAG1 delivery. For example, protein G has been used by many studies for indirect immobilization and they have demonstrated the functionality of JAG1 in activating the canonical NOTCH gene expression pathway (Osathanon et al., 2013; Manokawinchoke et al., 2017; Nowwarote et al., 2018; Chansaenroj et al., 2022). A 2009 study used self-assembled monolayers for indirect affinity immobilization and it also established the ability of SAMs to allow JAG1-induced NOTCH signaling activation (Goncalves et al., 2009). Both methods demonstrated that using this indirect strategy for lower JAG1 concentrations improved JAG1 immobilization and NOTCH activation *in vitro*. However, because these studies were *in vitro*, *in vivo* studies are needed to confirm the clinical translatability of this delivery method. Moreover, none of these studies demonstrated the release kinetics of JAG1 and rather focused on demonstrating that JAG1 immobilization was achieved. Dishowitz et al. (2014) compared two JAG1 immobilization strategies where JAG1 was immobilized indirectly and directly to an osteogenic biomaterial termed A6 to develop a new therapy that promotes bone tissue formation. The results found that the direct method increased the surface density of successfully immobilized JAG1 compared to the indirect method, therefore establishing it as the most viable strategy. Moreover, this was the first study to demonstrate that JAG1 delivery transiently activates the NOTCH signaling pathway. Chansaenroj et al. (2022) was the first study to perform a proteomic study on hDPSCs that were cultured with indirectly immobilized JAG1. Upon extracting the protein component of cells, it was found that JAG1 stimulated the expression of proteins associated with osteogenic differentiation and matrisome proteins played a role in osteogenic differentiation. The novelty of this study is the use of a proteomic study to demonstrate an interplay of JAG1 and ECM on osteogenic differentiation, suggesting the clinical translatability of this approach. Studies have also introduced protein G-coated dynabeads as a method to immobilize JAG1 and deliver it *in vitro* (Zhu et al., 2013; Ndong et al., 2018; Zohorsky et al., 2021) and *in vivo* (Kamalakar et al., 2021). Despite its efficiency in immobilizing JAG1 and its potential for translatability, these super paramagnetized spherical beads have the limitation of being toxic if used as a treatment for children. Their toxicity was observed by Tiwari et al. (2003) where they used these beads to improve cell extraction. It was found that using a large number of the dynabeads led to a reduction in endothelial cell proliferation and metabolism. Self-assembling peptide hydrogels have also been functionalized with peptide mimics of JAG1. The use of these biomaterials as a delivery method is appealing due to their ease of synthesis, low immunogenicity, and the ability to incorporate bioactive motifs in them. Boopathy et al. (2014) demonstrated that NOTCH activation was possible with such biomaterials, however, this activation was found to be dependent on hydrogel polymer density and there was an increase in activation in the presence of the active JAG1 mimic RJ. Moreover, this study focused on CPC delivery and their therapeutic benefits in a myocardial infarction rat model. Therefore, there is a need to clearly describe the effects of the SAP hydrogels that contain RJ (RJ-SAP) on endogenous CPCs for clinical translatability. Boopathy et al. (2015) examined these hydrogels in a cell-free setting and evaluated their effect of NOTCH activation on a rat MI model. Parameters indicative of cardiac functional improvement were found to be increased in rats treated with RJ-SAP hydrogels. Moreover, there was an increase in endothelial cell proliferation as well as vessel area, indicating an RJ-SAP-mediated NOTCH activation. However, this study could not determine whether the hydrogels effected proliferation of existing endothelial cells or stem cells differentiated to endothelial cells. To make these finding clinically translatable, analysis of the mechanism of the RJ-SAP hydrogels on different cell types in the heart should be performed. Additionally, the long-term effects of hydrogel delivery should be investigated. Finally, studies in a larger animal model of MI should be performed to determine the scale up of the hydrogels.

Nanoparticles can also be functionalized with JAG1 which is what Matea et al. (2015) performed to promote an efficient interaction between JAG1 and NOTCH. Gold nanoparticles were used because of their stability in aqueous media, size distribution, and their ability to react with a broad range of ligands. Direct and indirect methods were used to immobilize JAG1 to the gold nanoparticles, and it was found that the indirect method produced a more uniform and stable nanoparticle than the direct method did. However, like the other indirect affinity immobilization methods previously discussed, these nanoparticles were only applicable *in vitro*. While this study did focus on establishing a strategy that delivers JAG1, it did not assess the nanoparticle’s potential of activating the NOTCH pathway. Gonzalez-King et al. (2017) was able to package JAG1 in HIF-MSC-derived exosomes and demonstrate the ability of these exosomes to induce angiogenesis *in vitro* and *in vivo*. For the *in vitro* study, JAG1-containing exosomes from HIF-MSCs were cultured with Human umbilical vein endothelial cells (HUVECs). Although this study suggested a novel mechanism for JAG1 delivery, its major limitation was that it did not demonstrate whether angiogenesis induction was from the direct interaction between the MSC-derived exosomes and the NOTCH receptors located on the endothelial cell plasma membrane or if it was due to the transfer of JAG1 from the MSCs to the HUVECs. Therefore, each component of the system that contributes to angiogenesis must be assessed. Additionally, the study did not quantify the concentration of JAG1 present in the exosomes. Therefore, it is unclear how this approach would be clinically translatable. Quantifying JAG1 would provide a better understanding of dosing adjustments required for treatment, therefore making this approach clinically translatable.

Youngstrom et al. (2017) used microcarrier beads to immobilize JAG1 which were incubated with collagen grafts and delivered to calvarial defects in mice. Bone regeneration was observed in these studies; however, it was not determined if JAG1 was bound in an orientation that facilitated cell-to-cell signaling and it was not confirmed that JAG1 was tethered to these beads, rather it was encapsulated. Moreover, release kinetics of JAG1 was not demonstrated in this study and the mechanistic basis of the improved healing observed was not defined. Fibrinogen based microspheres were another method used to deliver JAG1. Manaspon et al. (2020) demonstrated that the JAG1-containing fibrinogen-based microspheres activated the NOTCH canonical pathway, however, in addition to this being an *in vitro* study, this study served as a proof of concept for the need to immobilize JAG1 and the use of microspheres for JAG1 delivery. Furthermore, these biomaterials would further need to be analyzed for their biological effects in the context of cell differentiation promotion. Finally, the binding capacity and conjugation of JAG1 to these microspheres would need to be scaled for clinical applicability.

Topical application of JAG1 dissolved in purified water as a treatment for hair loss was investigated by Lin et al. (2019) and they found that EGF and JAG1 have a synergistic effect on each other *in vivo*. However, further analysis will be needed to be performed on the mechanism of the hair growth promoting effect JAG1 and EGF has as it is unclear if this effect was due to the stimulation of cell proliferation. Moreover, there was no treatment group that was treated with JAG1 alone, without the presence of testosterone. As we know from literature, testosterone promotes hair growth (Glaser et al., 2012). Therefore, it would be difficult to conclude that JAG1 could promote hair growth in the absence of testosterone.

The studies discussed in this review all provide a proof of concept on novel methods that deliver JAG1 which can be used as a therapeutic. However, studies that rely on an indirect affinity immobilization method have the limitation of not being clinically applicable as they do not address the importance of ligand density in cell behavior regulation. Studies that did not use this method were successful in initiating NOTCH activation via JAG1, however the substrates used were proven to be toxic. Directly immobilizing JAG1 to PEG-MAL hydrogels without the use of dynabeads could be an alternative approach that can address the limitations of these studies. These hydrogels are desirable as regenerative scaffolds due to their tunable degradation ability, minimal inflammation, and binding ability (Ndong et al., 2018).

## 7 Conclusion

The NOTCH signaling pathway is involved in a variety of cell-fate decisions and JAG1 is a well-known ligand and activator of the NOTCH pathway, making it a target for therapeutic applications. However, an optimal delivery mechanism for JAG1 has yet to be established as there are two main requirements for the successful delivery of JAG1 and its successful activation of the NOTCH canonical pathway: 1) JAG1 must be immobilized to a delivery scaffold and 2) a correct orientation must be achieved between the NOTCH receptor at the cell surface and the JAG1 ligand of the other cell to ensure cell-to-cell contact. Many approaches have been proposed to address these requirements such as using an indirect affinity immobilization method where the delivery scaffold could be protein G, self-assembled monolayers, synthetic membranes, and nanoparticles. However, these delivery methods come with the limitation of not being clinically-applicable due to its low yield of ligand density which is vital for cell behavior regulation such as differentiation and proliferation. Other delivery scaffolds that have been suggested are dynabeads, self-assembling peptides, nanocarriers, cell-derived exosomes, collagen scaffolds, and microspheres. Some approaches, such as the use of dynabeads alone or encapsulating these dynabeads in PEG-MAL hydrogels, have been proven to be toxic, while other approaches lack the fundamental understanding of how the canonical pathway is activated. Therefore, there is a need to expand upon these delivery methods to make them clinically applicable. The use of PEG-MAL hydrogels to directly immobilize JAG1 without the use of a secondary substrate such as dynabeads can be an approach for future studies.

## Figures and Tables

**Table:** 

**JAG1**	JAGGED1
**MSC**	Mesenchymal stem cells
**CNC**	Cranial neural crest cells
**PCL/HA**	Polycaprolatone-incorporated hydroxyapatite
**NICD**	NOTCH intracellular domain
**NRR**	Negative regulatory region
**DSL**	Delta/Serrate/LAG-2
**EGF**	Epidermal growth factor
**CR**	Cysteine rich
**ALGS**	Alagille syndrome
**TOF**	Tetralogy of Fallot
**LLC**	Lewis lung carcinoma
**TRAMP**	Transgenic Adenocarcinoma of the Mouse Prostate
**AC**	Adenocarcinomas
**SC/ASCs**	Squamous cell/adeno-squamous carcinomas
**BMD**	Bone mineral density
**HPDLs**	Human periodontal ligament derived mesenchymal stem cells
**hDPs**	Human pulp cells
**CPC**	Cardiac progenitor cells
**Treg**	T regulatory cells
**HFSC**	Hair follicle stem cell
**IgG**	Immunoglobulin G
**REEC**	Rat esophageal epithelial cells
**ECM**	Extracellular matrix
**HCASMC**	Human coronary artery smooth muscle cells
**10T1/2**	Embryonic multipotent mesenchymal progenitor cells
**HEPM**	Human embryonic palatal mesenchyme
**PEG-4MAL**	Polyethylene glycol maleimide
**SAMs**	Self-assembled monolayers
**HL-60**	Leukemic cell line
**SAP**	Self-assembling peptides
**GNPs**	Gold nanoparticles
**HIF-1α**	Hypoxia inducible factor-1α
**FbMs**	Fibrinogen-based microspheres
**tFbMs**	Thrombin crosslinked fibrinogen microspheres
**HUVECs**	Human umbilical vein endothelial cells
